# NLRP3 inflammasome couples purinergic signaling with activation of the complement cascade for the optimal release of cells from bone marrow

**DOI:** 10.1038/s41375-019-0436-6

**Published:** 2019-03-07

**Authors:** Mariusz Z. Ratajczak, Mateusz Adamiak, Arjun Thapa, Kamila Bujko, Katarzyna Brzezniakiewicz-Janus, Anna M. Lenkiewicz

**Affiliations:** 1 Stem Cell Institute at James Graham Brown Cancer Center, University of Louisville, Louisville, KY USA; 2 Center for Preclinical Studies and Technology, Department of Regenerative Medicine Warsaw Medical University, Warsaw, Poland; 3 Department of Hematology, University of Zielona Gora, Hospital Gorzow Wlkp, Zielona Gora, Poland

**Keywords:** Immunopathogenesis, Cell signalling

## Abstract

The mechanisms that regulate egress of hematopoietic stem/progenitor cells (HSPCs) into peripheral blood (PB) in response to stress, inflammation, tissue/organ injury, or administration of mobilization-inducing drugs are still not well understood, and because of the importance of stem cell trafficking in maintaining organism homeostasis, several complementary pathways are believed to be involved. Our group proposes that mobilization of HSPCs is mainly a result of sterile inflammation in the bone marrow (BM) microenvironment in response to pro-mobilizing stimuli and that during the initiation phase of the mobilization process BM-residing cells belonging to the innate immunity system, including granulocytes and monocytes, release danger-associated molecular pattern molecules (DAMPs, also known as alarmins), reactive oxygen species (ROS), as well as proteolytic and lipolytic enzymes. These factors together orchestrate the release of HSPCs into PB. One of the most important DAMPs released in the initiation phase of mobilization is extracellular adenosine triphosphate, a potent activator of the inflammasome. As a result of its activation, IL-1β and IL-18 as well as other pro-mobilizing mediators, including DAMPs such as high molecular group box 1 (Hmgb1) and S100 calcium-binding protein A9 (S100a9), are released. These DAMPs are important activators of the complement cascade (ComC) in the mannan-binding lectin (MBL)-dependent pathway. Specifically, Hmgb1 and S100a9 bind to MBL, which leads to activation of MBL-associated proteases, which activate the ComC and in parallel also trigger activation of the coagulation cascade (CoaC). In this review, we will highlight the novel role of the innate immunity cell-expressed NLRP3 inflammasome, which, during the initiation phase of HSPC mobilization, couples purinergic signaling with the MBL-dependent pathway of the ComC and, in parallel, the CoaC for optimal release of HSPCs. These data are important to optimize the pharmacological mobilization of HSPCs.

## Introduction

Clinical data indicate that a significant number of donors of hematopoietic stem/progenitor cells (HSPCs) respond poorly to mobilization by granulocyte colony-stimulating factor (G-CSF) and CXCR4-blocking small-molecule AMD3100 [1–5]. Therefore, the optimization of stem cell mobilization and the subsequent enhancement of seeding efficiency of HSPCs in BM after transplantation are important goals to improve clinical outcomes of transplantations, and this will be addressed in this review. We will employ in this review for reasons of simplicity the term HSPCs as both stem and progenitor cells are present in the mobilization product; however mechanisms described herein may affect hematopoietic stem cells (HSCs) and hematopoietic progenitor cells (HPCs) in various ways. This, however, requires further studies.

It is well known that HSPCs are nonstop travelers throughout the body. During embryogenesis, they migrate between different organs that maintain active hematopoiesis, and later on in postnatal life, a small number continue to circulate in peripheral blood (PB) [1–5]. These HSPCs circulating under steady-state conditions patrol tissues and organs in search of damage and keep in balance the pool of stem cells, which is spread throughout the hematopoietic niches in remote areas of bone marrow (BM) tissue [1, 5–7]. This circulation of HSPCs undergoes circadian rhythm changes, in which more of these cells are present in PB in the early morning hours than late at night [8]. We have proposed that this process is related, at least partially, to changes in tonus of the complement cascade (ComC) and the coagulation cascade (CoaC), which are regulated by deep-sleep hypoxia [1, 9].

As is well known, HSPCs are retained in their BM niches by signals involving the interactions between (i) the α-chemokine receptor CXCR4 and (ii) an α4β1 integrin dimer composed of CD49d (alpha 4) and CD29 (beta 1), also known as very-late antigen 4 (VLA-4), present on the HSPC surface [7, 10, 11]. Both of these cell surface receptors, CXCR4 and VLA-4, interact with the corresponding ligands, stromal-derived factor 1 (SDF-1) and vascular cell-adhesion molecule 1 (VCAM-1), respectively, which are expressed by the cellular components of stem cell niches [1, 5, 7].

Pharmacological mobilization is a means to obtain HSPCs for hematopoietic transplantation and is induced by pro-mobilizing drugs. During administration of these drugs (e.g., G-CSF or AMD3100) the number of these cells in PB may increase up to 100-fold over steady-state conditions [1–7]. Mobilized HSPCs are subsequently harvested from PB by leukapheresis. The exact mechanisms that regulate this massive egress of HSPCs from BM into PB are still not well understood, and most likely several complementary mechanisms are involved [1–5, 12]. Our group proposes that pharmacological mobilization of HSPCs occurs as a result of “sterile” inflammation that is induced in the BM microenvironment in response to pro-mobilizing stimuli [1, 13, 14]. According to the definition, sterile inflammation is an inflammatory process that occurs in the absence of any microorganisms and during pharmacological mobilization is triggered by G-CSF or AMD3100 [15]. Sterile inflammation, like microbial-induced inflammation, is initiated by the activation of cellular elements of innate immunity, including neutrophils and macrophages, as well as by activation of the ComC [15].

Overall, we can divide the HSPC mobilization process into three principal phases: (i) initiation, (ii) amplification, and (iii) execution. In the (first) initiation phase, pro-mobilizing stimuli activate the cellular innate immunity network by stimulating BM-residing granulocytes and monocytes and perhaps also other innate immunity cells in the BM microenvironment to release danger-associated molecular pattern molecules (DAMPs), reactive oxygen species (ROS), and proteolytic and lipolytic enzymes [1, 3, 14–18]. As we recently demonstrated, an important role is also played by the activation of purinergic signaling, involving release of extracellular nucleotides (EXNs) from the activated cells [19]. The most important member of this class of molecules is adenosine triphosphate (ATP), a potent mediator in the extracellular purinergic signaling network [20]. These complementary signals allow HSPC detachment and egress from BM stem cell niches during the initiation phase of mobilization. What will be addressed in more details later on in this review is that the initiation phase leads to the activation of the ComC by triggering mannan-binding lectin (MBL)-dependent pathway of ComC activation. Next, during the amplification phase of mobilization, ComC becomes additionally activated by alternative activation pathway. Finally, in the execution phase of mobilization ComC final cleavage fragments C5 and C5a as well as activated CoaC are required for the optimal egress of HSPCs from BM into PB [14, 19].

**Fig. 1:**
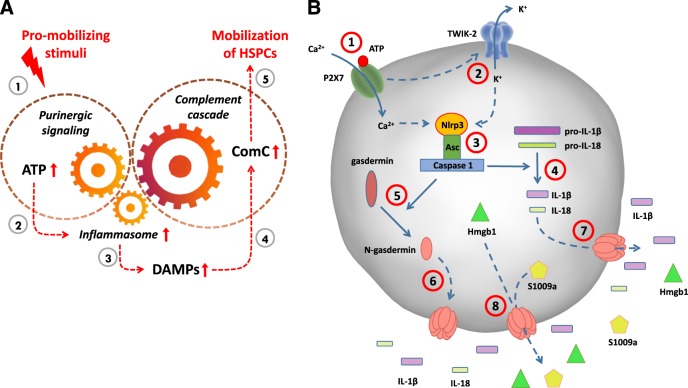
**a** The inflammasome as a gear or cogwheel that couples purinergic signaling with the complement cascade (ComC) in the process of HSPC mobilization. Pro-mobilizing stimuli (e.g., G-CSF) (1) release ATP from activated innate immunity Gr-1^+^ cells (2), which activates intracellular inflammasome via P2X7 purinergic receptors (3). As result of inflammasome activation several DAMPs are released, including Hmgb1 and S100a9 (4), which are recognized by MBL and activate the ComC in the mannan-binding lectin-dependent pathway. Activation of the ComC leads to release of C5 cleavage fragments that are crucial for optimal release of HSPCs from BM into PB (5). In parallel, what is not shown in this scheme, the CoaC is also activated in an MBL-dependent manner. **b** The most important steps in the intracellular activation of NRLP3 inflammasome by ATP: Extracellular ATP activates P2X7 (step 1), which subsequently activates K^+^ efflux channel TWIK2 (step 2). A decrease in K^+^ intracellular levels triggers the activation of the NRLP3 inflammasome complex (step 3). In response to this caspase, 1 cleaves pro-IL-1β and IL-18 to active ready for secretion IL-1β and IL-18 (step 4), and in addition cleaves gasdermin that releases *N*-gasdermin (Step 5) that insert into the cell membrane to create pores (step 6) for the release of IL-1β and IL-18 (step 7) as well as DAMPs (step 8)

In this review, we will present evidence from our recent investigations that during the initiation phase of mobilization, the ATP-activated inflammasome NLRP3 has an important role in activation of the ComC and CoaC. As depicted in Fig. 1a, the inflammasome has the role of a gear or cogwheel that couples activated purinergic signaling with activation of the ComC and CoaC. We will discuss this novel concept about the role of inflammasomes in the mobilization of HSPCs, as modulation of inflammasome activity may become an important means to improving HSPC yield during clinical pharmacological mobilization. The inflammasomes may also be involved in the egress of HSPCs into PB during other situations, such as systemic inflammation or stress related to tissue/organ injury, and it may also have a role in the circadian circulation of HSPCs in PB [21–23].

### The inflammasomes as important part of the innate immunity network

Inflammasomes are multiprotein oligomer complexes and important components of the innate immunity network that are triggered during “sterile” inflammation in response to DAMPs (also known as alarmins), which are released by activated or damaged cells, and during infections in response to pathogen-associated molecular pattern molecules (PAMPs) [21–23]. Here we will focus on the NLRP3 member of this family, which is highly expressed in innate immunity cells [21, 22].

The NLRP3 inflammasome is expressed by myeloid cells, including Gr-1^+^ granulocytes and monocytes, and is currently the best-characterized member of this family operating in hematopoietic cells [21, 22]. It is composed of several proteins, including NLRP3, CARD-containing adaptor (ASC), and caspase 1, and is responsible for the activation of inflammatory responses (Fig. 1b) [23]. Activation of intracellular caspase 1 promotes maturation and secretion of pro-inflammatory cytokines, such as interleukin 1β (IL-1β) and interleukin 18 (IL-18), which are activated inside cells by caspase 1 proteolytic cleavage of their pro-forms (pro-IL-1β and pro-IL-18) before release into the extracellular space [21–23]. This is facilitated by the creation of cell membrane pores owing to insertion into the membrane another caspase 1 cleavage product, *N*-gasdermin, that is, gasdermin protein fragment (Fig. 1b) [21–23].

It is known that the interleukin 1 family is composed of a group of 11 members, which include a complex network of pro-inflammatory cytokines that regulate and initiate inflammatory responses [24]. IL-1β, together with IL-1α, is the most-studied member of this cytokine family. In addition to granulocytes, monocytes, and macrophages, it is also secreted by dendritic cells, B lymphocytes, natural killer cells, microglia, fibroblasts, and epithelial cells [24]. IL-1β is synthesized after stimulation by DAMPs (e.g., ATP, Hmgb1, and S1000a9) and PAMPs (e.g., liposaccharide–LPS). In contrast to IL-1α, its expression is induced by the transcription factor NF-κB. Another member of the IL-1 family, IL-18, is also secreted as part of inflammasome activation by innate immunity cells. It has been reported that both IL-1β and IL-18 induce leukocytosis when injected into mice [25]. Besides pro-inflammatory properties both cytokines exert several other pleiotropic effects in hematopoiesis. For example, IL-1β may radioprotect adult BM HSPCs and increase their proliferation and differentiation [25]. On the other hand, IL-18 stimulates the secretion of several hematopoietic modulators including interferon-γ, IL-6, and granulocyte–macrophage colony-stimulating factor.

It is widely accepted that release of active IL-1β and IL-18 from cells is one piece of molecular evidence for activation of the inflammasome [21, 22]. However, in addition to these interleukins, upon activation of the inflammasome innate immunity cells release several other DAMPs, including Hmgb1 and S100a9, which are recognized by the PB-circulating collagen-containing C-type lectin (collectin) known as MBL, an important soluble member of the group of pattern-recognition receptors. Innate immunity cells also release ROS that expose neoepitopes on the surface of cells in the BM microenvironment, which after binding with naturally occurring IgM antibodies, also become targets for MBL [26]. ROS alone may also trigger activation of the inflammasome in innate immunity cells [21–23]. Based on these mechanisms, the NLRP3 inflammasome activates the MBL-dependent pathway of the ComC by cell-secreted DAMPs (e.g., Hmgb1 or S100a9) as well as ROS and also collaterally triggers another ancient proteolytic cascade, the CoaC. It is important to state, which in addition to NLRP3 inflammasome, other important members of this family including NLRP1 and NLRP12, which are also highly expressed in hematopoietic cells and respond to DAMPs [27, 28] may play similar role. Moreover, similarly, the potential involvement of NLRC4 and AIM2 inflammasomes in “sterile” inflammation of BM requires further studies [29].

Using appropriate knockout (KO) mice, we previously demonstrated that of the three pathways of ComC activation, (i) the C1q-mediated classical pathway, (ii) the MBL-mediated pathway, and (iii) the factor B (FB)-regulated alternative pathway, the MBL pathway of the ComC has the most important role in pharmacological mobilization of HSPCs [26]. In support of this conclusion, MBL-KO mice, which have a defective MBL pathway of ComC activation, and not C1q-KO mice, which do not activate the classical ComC pathway, are poor mobilizers [1, 30]. Our most current research also indicates involvement of the alternative ComC pathway, which, under steady-state conditions, is continuously auto-activated at a low level in PB and potentiates MBL-mediated activation of the ComC during mobilization. Specifically, FB-KO mice, which do not activate the alternative pathway of the ComC are, like MBL-KO mice, poor mobilizers (manuscript in preparation). This finding supports an active involvement of the alternative pathway of ComC activation during the amplification phase of the mobilization process.

The involvement of the NLRP3 inflammasome in mobilization of HSPCs is supported by our recent results, demonstrating that mice exposed to MCC950 inhibitor of the ASC that is a crucial component of NRLP3 inflammasome [21–23] become poor mobilizers (manuscript in preparation). What is also important to emphasize that, in addition to Hgmb1 and ROS released from myeloid cells owing to inflammasome activation, IL-1β and IL-18 released from innate immunity cells during inflammasome activation also increase on their own the egress of cells from BM. Both of these cytokines are strong pro-mobilizing mediators, and to corroborate this we confirmed the pro-mobilizing activity of IL-1β [25] and determined that another inflammasome-released cytokine, IL-18, is also a potent pro-mobilizing factor. Interestingly, although neither IL-1β nor IL-18 alone can chemoattract HSPCs, mice injected with these cytokines release a comparable number of HSPCs from BM as the administration of AMD3100. At this point, the molecular mechanism behind this phenomenon is unclear, but we propose that secreted IL-1β and IL-18, by means of an autocrine positive-feedback loop, potentiate activation of the NLRP3 inflammasome to release more Hmgb1 and ROS and thus amplify the MBL-mediated pathway of ComC activation. Supporting such a mechanism is the finding that IL-1β and IL-18 receptors are highly expressed by innate immunity cells.

It is well known that activation of the NLRP3 inflammasome requires two signals [21–23]. The first is a priming signal that is mediated by bacterial LPS activating Toll-like receptor 4 or by the TNF-α-activating TNF receptor. This first signal induces the NF-κB pathway, leading to upregulation of pro-IL-1β and NLRP3 protein levels and can also be amplified by autocrine-secreted IL-1β or IL-18 [21–23]. The second signal promotes the assembly of CARD-containing adaptor protein (ASC) and pro-caspase 1, leading to activation of the NLRP3 inflammasome complex [22]. What is important for the topic of this review, this second signal under noninfectious conditions, as seen for example during sterile inflammation, is provided by extracellular ATP, which leads to inflammasome activation by activating P2X7 purinergic receptors on the surface of innate immunity cells and thereby triggering potassium efflux [21–23]. Recently, an important role of the potassium efflux channel TWIK2 has been found in activation of the NLRP3 inflammasome [31].

In further support of a role for the inflammasome in the mobilization process, LPS, which provides the first priming signal for inflammasome activation, has previously been demonstrated in mice to facilitate the mobilization process, as mice exposed to antibiotics that remove LPS-producing Gram-negative bacteria form the intestine show a decrease in the number of mobilized HSPCs [32, 33]. On the other hand, defective release of ATP by pannexin [19] or connexin [34] channels or defective release in P2X7-KO mice, which do not respond to the ATP-mediated second signal, both result in poor mobilization in response to G-CSF. This result may be explained at the molecular level by the fact that ATP–P2X7 signaling opens the inflammasome-triggering TWIK2 potassium efflux channel [31]. What is also important is that activation of ComC activates NRLP3 and most likely NRLP1 inflammasome by direct stimulation of C3a and C5a receptors on innate immunity cells by C5a by C3 and C5 cleavage products as well as owing to increasing cell membrane permeability for potassium efflux by final mediator of ComC activation that is C5bC9 membrane attack complex (MAC) [21, 22]. This further support a crucial involvement of ComC activation as orchestrator of HSPCs mobilization [1, 11].

### ATP as a crucial mediator of purinergic signaling, activating the NLRP3 inflammasome

As is well known, primarily, intracellular ATP provides energy to drive many processes in living cells and is involved in energy transfer. In addition, when secreted from the cells, it becomes a crucial mediator in the extracellular purinergic signaling network [35]. Purinergic signaling is considered to be an evolutionarily ancient signaling mechanism that regulates several aspects of cell biology, including providing chemotactic signals and modulating the responsiveness of innate and acquired immune cells to inflammatory cues [19, 20]. Extracellular ATP binds to P2 purinergic receptors expressed on all cell types, including hematopoietic cells [35]. Based on their structural characteristics, P2 purinergic receptors are subdivided into metabotropic (P2Y) and ionotropic channel (P2X) receptors. The P2Y receptor family includes eight G protein-coupled receptors (P2Y1, 2, 4, 6, 11, 12, 13, and 14). By contrast, the P2X receptor family consists of seven ionotropic channel members (P2X1, 2, 3, 4, 5, 6, and 7), which, after stimulation by ATP, allow for efflux of K^+^ and influx of Ca^2+^ and Na^+^. The P2X7 receptor, which has a role in ATP-mediated NRLP3 inflammasome activation, belongs to this second family of receptors [35].

ATP, a major ligand for P2 receptors, is released from the cells by exocytosis or in a conductive mechanism that involves two types of plasma membrane maxi ion channels or pore-forming channels, including (i) pannexins and (ii) connexins [35]. Pannexins predominantly exist as large transmembrane channels connecting the intracellular and extracellular space, allowing the passage of ions and small molecules, including ATP, between these compartments. We have shown that inhibition of pannexin 1 with probenecid or pannexin blocking peptides [19] results in a decrease in ATP release from BM-residing innate immunity cells and, as a consequence, impairs G-CSF and CXCR4 antagonist (AMD3100)-mediated mobilization of HSPCs [19]. Like pannexins, certain connexins (e.g., Cx43) that are located to non-junctional regions of the cell membrane may also release ATP into the extracellular space. Supporting the role of CX43 connexin in extracellular release of ATP, it has been reported in an excellent paper that Cx43-KO mice are poor mobilizers [34].

Based on these findings, ATP secreted from G-CSF-activated neutrophils and monocytes as a DAMP molecule is involved in the induction of sterile inflammation and couples the purinergic signaling network with the ComC via the NLRP3 inflammasome (Fig. 1). Most important is its interaction with the P2X7 receptor on the surface of cells of the innate immunity system [19]. Interestingly, human loss or gain of function of P2X7 expression is differently influenced by the inheritance of various P2X7 single-nucleotide polymorphisms (SNPs). It has been reported that the Ala348Thr polymorphism results in a threefold increase in P2X7 activity over wild type [36]. Moreover, when Ala348Thr is co-inherited with Gln460Arg as a haplotype, the activity of P2X7 is further amplified to fivefold over those subjects not carrying this haplotype. In a recent report, it was demonstrated that the presence of the SNP Gln460Arg co-inherited with Ala348Thr, which leads to P2X7 hyperactivity, results in a significant increase in HSPC mobilization [36]. This result supports the important role of the ATP–P2X7–NLRP3 inflammasome axis in this process.

ATP, however, also activates several P2Y G protein-coupled purinergic receptors in parallel, and the exact involvement of these receptors in HSPC mobilization requires further study [37]. Nevertheless, a significant role seems to be played here by ATP-mediated upregulation of phospholipase β2 (PLC-β2) in hematopoietic cells [37]. Specifically, PLC-β2, which is primarily involved in the intracellular signaling cascade, when released from activated granulocytes during degranulation digests the glycolipid glycosylphosphatidylinositol anchor, which plays a crucial role in maintaining the integrity of lipid rafts on HSPC extracellular membranes [1, 11]. As mentioned above, lipid rafts contain CXCR4 and VLA-4 receptors, which are crucial for anchoring HSPCs to their respective ligands SDF-1 and VCAM-1 in their BM niches [1–5, 11]. Given this mechanism, ATP involvement in the initial phase of mobilization of HSPCs is based both on activation of the inflammasome and on the parallel release of PLC-β2 from neutrophils [37]. The importance of granulocytes in the mobilization process has been reported in the past and can be understood [38] in light of recent observations by their (i) involvement in triggering inflammasome activation and (ii) the release of proteolytic and lipolytic enzymes [21, 22, 39].

**Fig. 2:**
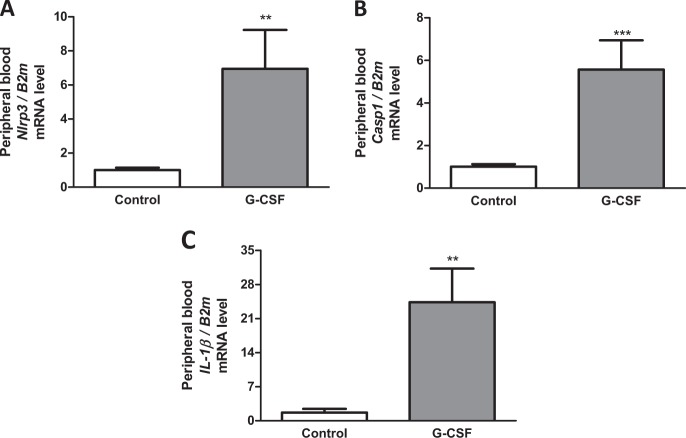
Molecular evidence of inflammasome activation in response to pro-mobilizing agents. Expression of Nlrp3 **a**, Casp 1 **b**, and IL-1β **c** genes in mice peripheral blood after 72-h treatment with cytokine granulocyte colony-stimulating factor (G-CSF; three injections, 100 μg/kg per day) measured by qRT-PCR. Results were normalized to beta-2-microglobulin (B2m) level. Data represent the mean value ± SEM for four independent experiments. **p < 0.01; ***p < 0.001 (Student’s *t* test)

In summary, activation of the NLRP3 inflammasome releases (i) IL-1β and IL-18, which results in its auto-activation by an autocrine positive-feedback loop, and (ii) several DAMPs, such as Hmgb1 and S100a9, which are recognized by MBL circulating in PB. DAMP–MBL complexes activate mannan-associated proteases (MASPs), which cleave/activate both C3 and prothrombin, which in turn induce activation of the ComC and CoaC [26]. Of note, the levels of IL-1β, IL-18, Hmgb1, and S100a9 increase in biological fluids during sterile inflammation, and this is seen during HSPC mobilization. Fig. 2 demonstrates induced expression of mRNA encoding Nlrp3, Casp 1, and IL-1β in PB mononuclear cells in mice mobilized for 3 days by G-CSF.

We have also identified in the past two important inhibitors of HSPC mobilization: (i) heme oxygenase 1 (HO-1) [40] and (ii) inducible nitric oxide synthase (iNOS) [41] (Fig. 2). Both of these enzymes have anti-inflammatory activity, and both inhibit release of HSPCs from BM into PB. What is important for the topic of this review, both HO-1 and iNOS have been reported to be NLRP3 inflammasome inhibitors [42–44].

In the extracellular space, ATP is processed as a purinergic mediator by the cell surface-expressed ectonucleotidases CD39 and CD73 to its metabolites ADP and AMP (products of CD39) and adenosine (product of CD73) [35]. Of note, we reported that adenosine, in contrast to ATP, inhibits mobilization of HSPCs [19]. This occurs owing to adenosine-mediated (i) upregulation of HO-1 and iNOS in HSPCs and granulocytes, which directly inhibits cell migration, (ii) direct inhibition of the inflammasome in innate immunity cells, and (iii) inhibition of the degranulation of granulocytes in the initiation phase of mobilization. Most importantly, adenosine activates the P1 family of G protein-coupled purinergic receptors (A1, A2A, A2B, and A3). As we have demonstrated, inhibition of the CD39 and CD73 ectonucleotidases, which process the degradation of ATP to adenosine in the extracellular space, enhances the mobilization of HSPCs [45]. Thus, as follow-up of this data we are currently investigating which of the P1 receptors is responsible for the mobilization-inhibitory effects of adenosine.

**Fig. 3:**
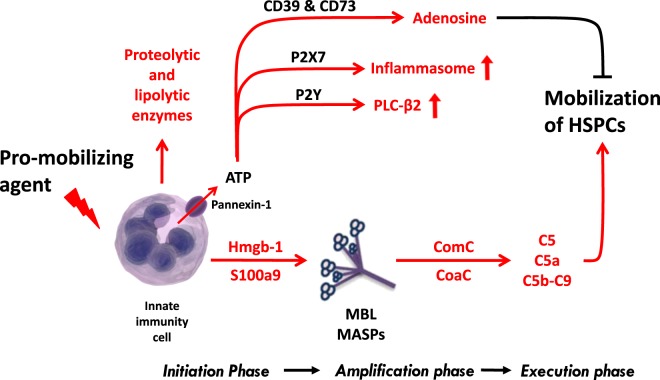
The interplay between purinergic signaling and ComC activation during mobilization of HSPCs. Pro-mobilizing agents (e.g., G-CSF) activate innate immunity cells (e.g., granulocytes or monocytes) to secrete proteolytic and lipolytic enzymes as well as several DAMPs, including ATP, Hmgb1, and S100a9. ATP is a potent activator of the inflammasome, which potentiates, through the P2X7 receptor, the release of HMGB1 and S100a9 from innate immunity cells, and stimulates via P2Y receptors the degranulation of neutrophils, which release more PLC-β2 and proteolytic enzymes. In the next step, HGMB1 and S100a9 proteins activate the complement cascade (ComC) in the MBL-dependent pathway, and PLC-β2 disrupts lipid rafts on the surface of HSPCs, which play a role in the retention of HSPCs in BM stem cell niches. Thus, both DAMPs and PLC-β2 promote effective mobilization. At the same time, ATP is processed to adenosine by CD39 and CD73 ectonucleotidases, which inhibits the mobilization process by (i) upregulating heme oxygenase 1 (HO-1) and inducible nitric oxide synthetase (iNOS) in HSPCs and innate immunity cells and (ii) inhibiting the degranulation of neutrophils. The ComC, together with the CoaC, is activated in the MBL-dependent pathway, which releases C5 cleavage fragments, which are in turn crucial in inducing the egress of HSPCs from BM into PB. Pathways promoting mobilization are shown by red arrows and the adenosine inhibitory pathway by a black arrow. There are also indicated three phases of mobilization—initiation phase (activation of NRLP3 inflammasome and release of DAMPs activating the MBL pathway of ComC), amplification phase (NRLP3 products and alternative ComC activation pathway amplify sterile inflammation in BM), and the execution phase (ComC cleavage fragments and activation of CoaC permeabilize BM–PB barrier to facilitate the egress of HSPCs). (adapted from Adamiak M. et al. Oncotarget, 2018, Vol. 9, (No. 90), pp: 36052-36054)

Figure 3 illustrates the general scheme of HSPC mobilization, depicting the promoting effect of ATP and the inhibitory effect of adenosine on the egress of HSPCs from BM into PB. It also shows the crucial involvement of Gr-1^+^ cell-released ATP in response to mobilizing agents on activation of the inflammasome and the release of several DAMPs and degranulation of neutrophils to release PLC-β2. DAMPs (Hmgb1 and S1009a) released during inflammasome activation trigger activation of the ComC and CoaC in an MBL–MASP-dependent manner. The scheme does not show the release of IL-1β and IL-18, which have a role in positive-feedback activation of the inflammasome.

In addition to hematopoietic cells, both EXNs and purinergic signaling modulate the function of other BM components, including mesenchymal cells and endothelial cells, and their impact on these cells during mobilization requires further study. Further work is also required to see whether other pro-mobilizing stimuli, such as growth-regulated protein beta [46], are also involved in purinergic signaling-initiated HSPC mobilization. It would be also interesting to see if inflammasomes in addition to HSPCs also affect mobilization of other BM-residing stem cells [47–49].

### Activation of the ComC and its importance for optimal release of HSPCs from BM in the execution phase of mobilization

As mentioned above, mobilizing agents induce a cascade of events in the BM microenvironment that can be considered “sterile” inflammation [14, 19]. However, like microbial-induced inflammation, this state is marked by the activation of cellular and soluble elements of innate immunity, including neutrophils and macrophages, as well as the ComC. We have already reported the crucial role of the MBL–MASP pathway of ComC activation in the initiation phase of mobilization [26]. In further support of this finding, mice that are deficient in MBL or MASP-1 are poor mobilizers in response to G-CSF and AMD3100.

Next, ComC activation and release of C5 cleavage fragments has a crucial role in the execution phase of mobilization, and C5 is cleaved by classical C5 convertase, which is generated during activation of the proximal part of the ComC. In further support of this mechanism, we have demonstrated that mice deficient in the C5 component of the ComC are poor mobilizers in response to G-CSF and AMD3100 [50, 51]. In addition, “C5-like” convertase activity is also provided by thrombin as a result of CoaC activation [52]. Interestingly, inhibition of thrombin by refludan also has a negative effect on the release of HSPCs, which supports the existence of a proteolytic interplay between the two ancient proteolytic cascades [4, 12]. As it is shown in Fig. 3 inflammasome activates via MBL–MASP pathway both cascades.

Activation of the distal part of the ComC during the execution phase of mobilization and the resulting release of C5 cleavage fragments promotes egress of HSPCs by several mechanisms. The C5 cleavage fragments, C5a and _desArg_C5a, are generated both in the BM microenvironment and in BM–blood sinusoids [1, 50]. Primarily, they both activate innate immunity cells in the BM microenvironment by positive feedback and enhance the state of sterile inflammation. Secondarily, although C5 cleavage fragments alone do not chemoattract HSPCs, they are potent chemoattractants for granulocytes and monocytes [1, 37, 50]. Therefore, the C5a and _desArg_C5a present in BM sinusoids create a gradient across the BM–blood endothelial barrier for egress of granulocytes and monocytes, and these cells enriched for proteolytic enzymes pave the way for HSPCs to leave BM by “following in their footsteps”. HSPCs then respond to the gradient of bioactive phosphosphingolipids (sphingosine-1-phosphate [S1P] and ceramide-1-phosphate [C1P]), which is already high, even under steady-state conditions in PB [1, 17, 53, 54]. Thus, after release from their niches HSPCs migrate to the BM sinusoids.

The important role of S1P and C1P in the egress of HSPCs is supported by the fact that both of these phosphosphingolipids create strong chemotactic gradients for HSPCs across the BM–PB endothelial barrier under steady-state conditions [17]. The fact that HSPCs released from their niches follow S1P and C1P gradients in PB indicates an active retention process for HSPCs in BM niches that counteracts these egress-promoting gradients. In support of this conclusion, the egress of HSPCs from BM is impaired in mice that have low levels of S1P in PB owing to sphingosine kinase 1 deficiency and, by contrast, is enhanced in mutant mice with sphingosine 2 kinase deficiency, which, surprisingly, have elevated levels of S1P in their PB plasma [17, 55]. Moreover, in addition to soluble C5a and _desArg_C5a, C5 cleavage leads to generation of membrane attack complex (MAC, also known as C5b-C9). MAC may additionally increase S1P levels in PB by inducing its release from erythrocytes, as these cells are highly enriched in this bioactive phosphosphingolipid [53].

Nevertheless, even strong upregulation of the S1P level in PB, as seen for example during phenylohydrazine-induced hemolysis, will not mobilize HSPCs as long as they are anchored in BM niches. Robust mobilization occurs first after co-administration of AMD3100 [56], which indicates active retention of HSPCs in BM niches and the need to first decrease this retention of HSPCs in BM niches to enhance mobilization in response to S1P gradient [56].

### Novel potential strategies to enhance mobilization of HSPCs

HSPCs mobilized into PB are easily isolated by leukapheresis, and they quickly engraft after transplantation. Thus, the pharmacological mobilization of HSPCs is a convenient strategy to obtain these cells for BM reconstitution after hematopoietic transplantation. Because some patients are poor mobilizers in response to conventional pro-mobilizing compounds (e.g., G-CSF), more efficient strategies are needed to obtain these cells for clinical purposes [1–5]. Based on the results presented in this review, it is reasonable to evaluate the efficacy of modulators of purinergic signaling, the inflammasome, and the ComC in enhancing this process.

As discussed, and as presented in Fig. 3, it is important to inhibit activity of the cell surface ectonucleotidases CD39 and CD73 to inhibit extracellular degradation of ATP to adenosine, which, as we have demonstrated, possesses anti-mobilizing properties [19, 45]. There are small-molecule inhibitors of these enzymes available, and our preliminary results in a murine model indicates that they could be promising pro-mobilizing adjuvant compounds if employed with G-CSF or AMD3100 [45]. Taking into consideration the important role of the inflammasome, one could consider application of ATP as a therapy to activate the inflammasome [19]. Our preliminary results in a murine model confirmed that ATP, together with G-CSF or AMD3100, enhances mobilization efficacy [19]. It is also possible that other activators of the inflammasome, such as the antibiotic nigericin or other reagents opening potassium efflux channels [31], could find similar application as well. We are currently testing this possibility in animal models.

Other potential strategies to enhance mobilization are related to optimal and adequate activation of the ComC. As has been demonstrated by us and others, activation of the ComC is negatively regulated by HO-1 and iNOS [40–44]. Therefore, small-molecule inhibitors of HO-1, as already shown in a murine model, could enhance mobilization of HSPCs if administered together with pro-mobilizing factors, as mentioned above [40]. This possibility is supported by the fact that HO-1 and iNOS are potent inhibitors of NLRP3 inflammasome activation [42, 43].

### Future directions in studying the role of the NLRP3 inflammasome in normal hematopoiesis

**Fig. 4:**
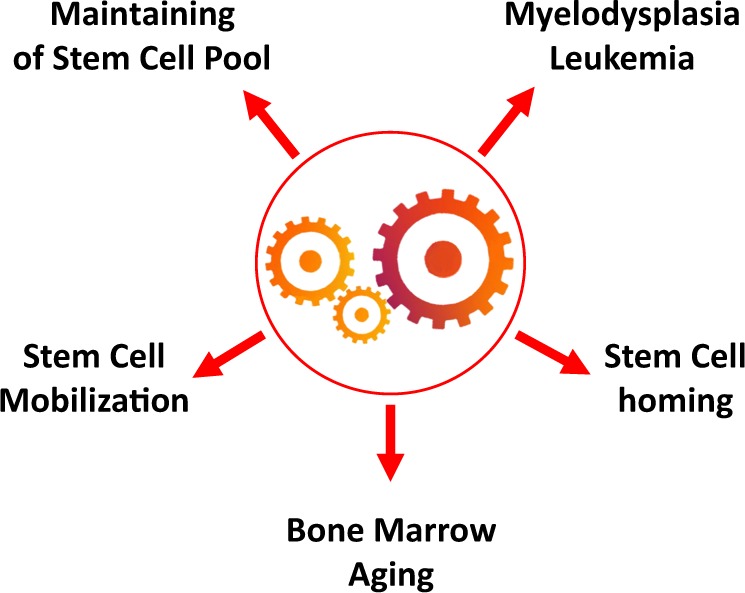
The proposed involvement of the NRLP3 inflammasome in regulating hematopoiesis. The inflammasome (shown as interacting gears/cogwheels) couples purinergic signaling with the ComC, which may affect several processes in BM, including stem cell mobilization, stem cell homing and engraftment, maintaining the pool of HSPCs, regulating aging, and playing a role in myelodysplasia and, as a consequence, in the origin of leukemia

The potential pleiotropic effects of the NLRP3 inflammasome on hematopoiesis are depicted in Fig. 4. In addition to the role of the NLRP3 inflammasome in the mobilization of HSPCs, further studies are needed to see whether it is also involved in the homing of HSPCs to BM. To justify this concept, we have demonstrated in the past that activation of the ComC in the BM microenvironment after conditioning for transplantation may have a role in accelerating hematopoietic reconstitution after HSPC transplantation [57]. Providing further support for this concept, by employing the Transwell system we demonstrated that, in addition to SDF-1, S1P, and C1P, ATP is a chemoattractant for HSPCs and thus a potential candidate homing factor for HSPCs [58].

To assess the role of ATP in homing and engraftment, we transplanted HSPCs from CXCR4^fl/fl^ Cre^Tg/–^ mice, in which CXCR4 had been eliminated by a Cre hematopoietic driver strategy into sphingosine kinase 1-deficient (Sphk1^–/–^) mice, which are deficient for S1P expression in BM. These experiments in mice, based on two defective homing axes, SDF-1–CXCR4 and S1P–S1P1 receptor, demonstrated the involvement of still other factor/s in the homing process, and ATP became the most likely candidate [58]. Specifically, ATP is released from cells in the BM microenvironment during conditioning for transplantation, and in addition to SDF-1 and S1P, could be involved as a homing chemoattractant for HSPCs. We also recently observed that exposure of HSPCs to a small-molecule inflammasome inhibitor before transplantation into lethally irradiated mice affects their homing and engraftment. Thus, the ATP-stimulated inflammasome has here a significant role (manuscript in preparation). In further support for a role of the inflammasome in HSPC homing and engraftment, our most recent results revealed that HSPCs exposed before transplantation to a P2X7 receptor antagonist, which prevents ATP-mediated activation of the inflammasome, also engraft more poorly than normal HSPCs.

A breakthrough in NLRP3 inflammasome research came from the observation that inflammasomes can also be released by macrophages as extracellular oligomeric particulate complexes. These particulate complexes may activate caspase 1 in the extracellular milieu, amplify inflammation, and be internalized by bystander cells and thereby contribute to the spread of the inflammatory reaction to these cells [21–23]. Moreover, we postulate that this phenomenon could involve extracellular microvesicles [59]. How important this intriguing phenomenon is in HSPC mobilization requires further study.

Another important aspect to investigate is the potential role of ATP-mediated inflammasome activation in regulating the pool of HSPCs via IL-1β. Inflammasome-mediated IL-1β signaling appears to serve as an integrator of metabolic activity downstream of ROS–HIF1α to promote HSPC formation and the development of the myeloid and lymphoid lineages in vivo and in vitro [60]. ATP that is secreted by cells in the BM microenvironment could have a role as an NLRP3 inflammasome activator to regulate IL-1β expression and thus have a role in regulating the pool of HSPCs.

Another important approach is to study the role of the inflammasome directly in hematopoietic niches. As of today, the hematopoietic stem cell niche remains incompletely defined and is described by competing models. This niche most likely is perivascular (SDF-1^+^ and KL^+^), created partially by mesenchymal stromal cells and endothelial cells, and is often, but not always, located near trabecular bone in the BM microenvironment [5, 61, 62]. Although HSCs are located around perivascular cells, early lymphoid progenitors are associated with the osteoblastic niche. The effect of ATP–inflammasome signaling in maintaining the integrity of these niches under steady-state conditions and during mobilization requires further study. Such investigations may also be relevant to better understanding the process of HSPC aging and the pathogenesis of myelodysplastic syndromes, in which the NRLP3 inflammasome has a role [63, 64].

Finally, after crossing a threshold of activation, the NRLP3 inflammasome may induce a form of cell death known as pyroptosis [21–23]. In this context, there are other members of this NLRP protein family, such as NLRP2, NLRPC3, NLRP6, NLRP7, NLRP10, NLRP12, and NLRX1, which have been suggested as playing inhibitory roles during inflammation by controlling caspase 1-mediated IL-1β secretion or by suppressing NF-κB signaling [21–23]. It would be interesting if these particular inflammasomes inhibit the effects of other pro-inflammatory inflammasomes in preventing stem cell mobilization, aging, and myelodysplasia

## Conclusions

We claim that innate immunity-mediated sterile inflammation induced in the BM microenvironment by pro-mobilization stimuli activates release of ATP from innate immunity cells. By involving P2X7 receptors, this release activates the NLRP3 inflammasome, which has the role of a “cogwheel” coupling purinergic signaling with activation of the ComC. This coupling is necessary to ensure optimal release of HSPCs from BM. In support of this notion, the NLRP3 inflammasome becomes activated during mobilization of HSPCs, and inhibition of its crucial component ASC results in a decrease in this process. Beside its role in mobilization of HSPCs, the NLRP3 inflammasome is also involved in maintaining the pool of HSPCs in BM niches. We are currently studying its role in the homing and engraftment of HSPCs after transplantation. As it has been reported that, besides the NRLP3 inflammasome, the NLRP1 and NLRP12 inflammasomes are expressed by hematopoietic cells, further study is needed to address whether they are also involved in HSPC trafficking. In support of this possibility, ATP activates the NLRP1 inflammasome by interacting with the P2X4 receptor in some cells. Moreover, some recent data indicate that NLRC4 and AIM2 inflammasomes are may be also activated be factors released during “sterile” inflammation [29] and their potential involvement in HSPCs mobilization clarification. Finally, further work should also address the role of NLRP family members (NLRP2, NLRC3, NLRP6, NLRP7, NLRP10, NLRP12, and NLRX1) in inhibiting inflammation [21–23, 27–29, 65], as they could have a negative role in the release of HSPCs from BM.
